# Motor properties of Myosin 5c are modulated by tropomyosin isoforms and inhibited by pentabromopseudilin

**DOI:** 10.3389/fphys.2024.1394040

**Published:** 2024-03-28

**Authors:** András Kengyel, Philip M. Palarz, Jacqueline Krohn, Anja Marquardt, Johannes N. Greve, Robin Heiringhoff, Anne Jörns, Dietmar J. Manstein

**Affiliations:** ^1^ Institute for Biophysical Chemistry, Hannover Medical School, Hannover, Germany; ^2^ Department of Biophysics, University of Pécs Medical School, Pécs, Hungary; ^3^ Institute of Clinical Biochemistry, Hannover Medical School, Hannover, Germany

**Keywords:** Myosin 5c, tropomyosin, pentabromopseudilin, molecular docking, immunohistochemistry

## Abstract

Myosin 5c (Myo5c) is a motor protein that is produced in epithelial and glandular tissues, where it plays an important role in secretory processes. Myo5c is composed of two heavy chains, each containing a generic motor domain, an elongated neck domain consisting of a single α–helix with six IQ motifs, each of which binds to a calmodulin (CaM) or a myosin light chain from the EF–hand protein family, a coiled–coil dimer–forming region and a carboxyl–terminal globular tail domain. Although Myo5c is a low duty cycle motor, when two or more Myo5c–heavy meromyosin (HMM) molecules are linked together, they move processively along actin filaments. We describe the purification and functional characterization of human Myo5c–HMM co–produced either with CaM alone or with CaM and the essential and regulatory light chains Myl6 and Myl12b. We describe the extent to which cofilaments of actin and Tpm1.6, Tpm1.8 or Tpm3.1 alter the maximum actin–activated ATPase and motile activity of the recombinant Myo5c constructs. The small allosteric effector pentabromopseudilin (PBP), which is predicted to bind in a groove close to the actin and nucleotide binding site with a calculated ΔG of −18.44 kcal/mol, inhibits the motor function of Myo5c with a half–maximal concentration of 280 nM. Using immunohistochemical staining, we determined the distribution and exact localization of Myo5c in endothelial and endocrine cells from rat and human tissue. Particular high levels of Myo5c were observed in insulin–producing β–cells located within the pancreatic islets of Langerhans.

## Introduction

Class V myosins belong to one of the ancient groups of the myosin superfamily, whose presence is essential from yeast to humans (Richards and Cavalier–Smith, 2005). The main function of class V myosins is to transport different cargoes such as organelles, vesicles or mRNA (Krementsova et al., 2017; Dolce et al., 2020). Class V is one of the three myosin classes, that can dimerize through the coiled–coil domain, but unlike class II myosins, members of class V do not form filaments and function exclusively as dimeric motors (Baboolal et al., 2009; Peckham, 2011).

Three isoforms of myosin V have been identified in vertebrates, which are produced and localized in different tissues (Rodriguez and Cheney, 2002). Myo5a, the most extensively studied member of this class, is highly produced in melanocytes and neurons, fulfilling a critical role in the trafficking of membranous organelles, including melanosomes in skin melanocytes and endoplasmic reticulum in Purkinje neurons (Rodriguez and Cheney, 2002; Jacobs et al., 2009; Hammer and Sellers, 2012; Gunther et al., 2014). Mutations in Myo5a lead to Griscelli syndrome, a disease characterized by hypopigmentation and neurological deficits (Rodriguez and Cheney, 2002; Gele et al., 2009; Dolce et al., 2020). Myo5b is produced in various tissues, where it functions in the recycling and transport of cytoplasmic vesicles and numerous receptors, such as the transferrin receptor or CXCR2 chemokine receptor (Takagi et al., 2008; Jacobs et al., 2009; Gunther et al., 2014). Additionally, Myo5b is produced in the intestine and its loss has been linked to microvillus atrophy and microvillus inclusion disease (Thoeni et al., 2014; Dolce et al., 2020). The third isoform of the myosin V class, myosin 5c, was discovered in 2002 through the analysis of the human EST (expressed sequence tags) database (Rodriguez and Cheney, 2002). Myo5c is widely distributed and most prominently produced in cells from intestinal and glandular tissues, including the colon, pancreas, mammary, thyroid, and different salivary glands. Myo5c is also present in various cell lines such as HeLa, HEK 293, Caco–2, or HUVEC, suggesting that Myo5c has a wide distribution among different cell types (Rodriguez and Cheney, 2002; Holthenrich et al., 2022). In endothelial cells Myo5c is involved in the transport and membrane fusion of Weibel–Palade bodies containing von Willebrand factor, an important hemostatic factor (Dolce et al., 2020; Holthenrich et al., 2022). Multi–ciliated cells possess a large number of motile cilia that beat in a coordinated and polarized manner. Myo5c was shown to control apical positioning of basal bodies in these cells that drive fluid flow in diverse tubular organs and are essential for the development and homeostasis of the vertebrate central nervous system, airway and reproductive tracts (Tu et al., 2017).

The three myosin V isoforms share a common four–domain structure. A generic motor domain binds actin in an ATP–dependent manner, an elongated neck domain providing binding sites for light chains and actin, a stiff lever arm, a coiled–coil domain (CCD) promoting the dimerization of two heavy chains, and a globular tail binding domain (GTD) that recognizes cargos or cargo adaptors (Reck–Peterson et al., 2000; Krementsov, Krementsova, and Trybus, 2004; Wang et al., 2004; Gunther et al., 2014). Adaptor proteins, such as small GTPases of the Rab family, interact with the GTD, thereby attaching class V myosins to their vesicular cargo (Zhang et al., 2018). The GTD of Myo5c interacts with Rab32, Rab38 or Rab3a resulting in attachment to secretory vesicles or melanosomes (Bultema et al., 2014; Dolce et al., 2020; Holthenrich et al., 2022).

Myo5a and Myo5b can adopt a triangular closed state structure with cargo binding and adenosine triphosphatase activity inhibited. The blocked ATPase activity and the cargo–/calcium–mediated activation of Myo5a and Myo5b is mediated through intra–and interpolypeptide chain interactions involving the motor domain, CCD and GTD of the heavy chain and CaM (Niu et al., 2022). Myo5c has an overall sequence identity of ∼50% with Myo5a or Myo5b. Sequence identity is highest in the motor domain region, whereas it reaches only 20%–30% in the coiled–coil region. Moreover, it lacks the PEST domain that is present in the other isoforms (Rodriguez and Cheney, 2002; Takagi et al., 2008). A number of structural changes in the region connecting the lever arm region to the CCD suggest that Myo5c has a different hinge structure to Myo5a, which may affect how, or whether, it forms a triangular closed–state structure. Alterations include a shortened or degenerated IQ6, a missing hinge strand, and differences in the sequence linking the N–terminal portion of the CCD (Niu et al., 2022).

Kinetic analysis of Myo5c revealed significant differences compared to the other vertebrate myosin V members. Myo5c functions as a low duty ratio motor. The rate–limiting step is phosphate release (Takagi et al., 2008). Myo5c does not move processively on actin filaments and its average velocity is an order of magnitude lower than that of Myo5a or Myo5b (Takagi et al., 2008). The low duty ratio suggests that Myo5c may act as a cargo transporter in the context of ensembles only or perform other functions, such as tethering organelles or generating tension (Krendel and Mooseker, 2005; Takagi et al., 2008).

Myosin light chains are essential for the proper functioning of myosins. These light chains belong either to the CaM or CaM–related gene families and are associated with the IQ motif located in the neck region of the myosin. According to previous research, the standard view has been, that unconventional myosins bind CaM, while conventional myosins bind essential light chains (ELC) and regulatory light chains (RLC) (Heissler and Sellers, 2014). Recombinant production of functional unconventional myosins in cells that overproduce CaM has demonstrated that CaM is able to bind these myosin heavy chains. However, it remains to be shown that CaM is the only native light chain (Heissler and Sellers, 2014). Recombinant Myo5b and Myo5c were purified and produced in functional form with CaM alone (Takagi et al., 2008; Watanabe et al., 2008; Yao et al., 2016; Heissler et al., 2017). In recent years, a number of findings have lent support to the hypothesis that light chains other than CaM can bind to unconventional myosins and thereby influence their activity and stability. For example, in the case of myosin VII and myosin XV, the production of a stable holoenzyme is facilitated by the co–purification of the unconventional myosin heavy chains with light chains other than CaM (Bird et al., 2014; Holló et al., 2023). While Myo5a extracted from mouse brain was purified exclusively with CaM, Myo5a from chicken brain also contained co–purified ELC (Wang et al., 2000).

Tropomyosins (Tpm) are important actin binding proteins, that stabilize the actin filament upon binding and modulate the interaction of the filament with other actin binding proteins, e.g., with myosins (Gunning et al., 2005; Manstein et al., 2020). They are important for a variety of actomyosin–dependent processes such as cell migration, cytokinesis, embryogenesis, and endo–or exocytosis (Curthoys et al., 2014; Kee et al., 2015; Wolfenson et al., 2016; Pathan–Chhatbar et al., 2018). The actin cofilaments with different Tpm isoforms were shown to modulate the motor properties of cytoskeletal myosins of classes I, II, and V. In addition, the N–terminal acetylation status of the Tpm isoforms was shown to contribute to the extent of modulation in an isoform–specific manner (Reindl et al., 2022). Tpm3.1 increases the sliding velocity of skeletal muscle myosin II on actin, whereas it has no effect on Myo5a (Barua et al., 2014; Barua et al., 2018). Tpm3.1 has also been shown to be involved in the regulation of organ size and cell proliferation in mice (Schevzov et al., 2015). The processive behavior of non–muscle Myo2a and Myo2b has been shown to be promoted by Tpm1.8 (Hundt et al., 2016; Pathan–Chhatbar et al., 2018). Tpm1.8 and Tpm3.1 lead to an increase in the ATPase activity of non–muscle Myo2b (Pathan–Chhatbar et al., 2018). Tpm1.6 was found in contractile stress fibers, where it is involved in their stabilization (Tojkander et al., 2011).

In this study, we show strong localization of Myo5c in human and rat pancreatic β–cells and in other glandular tissues. In addition, we report the purification and characterization of a heavy meromyosin (HMM) fragment of the Myo5c heavy chain that was either co–produced with CaM alone or with CaM, the ELC Myl6 and the RLC Myl12b. We describe the steady–state kinetics and motor function of the recombinant Myo5c constructs for the interaction with F–actin and selected cofilaments of actin with human cytoskeletal Tpm isoforms. In addition, the interaction of human Myo5c with PBP, a small allosteric effector of class V myosins, is described.

## Materials and methods

### Protein production and purification

The coding sequence of human Myo5c, which encodes the amino–acids 1–1108 (Uniprot–ID: Q9NQX4) (Myo5c–HMM) was fused to a C–terminal Flag affinity tag and cloned into the multiple cloning site of the pFastBac™ Dual expression vector under the control of the polyhedrin promotor (pFastBac–Myo5cHMM). pFastBac™ Dual vector containing CaM (Uniprot–ID: P0DP23) or the ELC and RLC (Myl6: Uniprot–ID: P60660 and Myl12b: Uniprot–ID: O14950) were prepared as described earlier (Reindl et al., 2022). Recombinant Myo5c–HMM was co–produced with CaM [Myo5c–HMM (CaM)] or with all three light chains: CaM, RLC and ELC [Myo5c–HMM (3LC)] in the baculovirus*/Sf9* system. Suspension cultures were harvested after 72 h and stored at −80°C.

For purification, cells were lysed in extraction buffer [10 mM MOPS–NaOH (pH 7.3), 200 mM NaCl, 15 mM MgCl_2_, 1 mM EGTA, 0.25 mM EDTA, 5 mM ATP, 0.1 mM DTT, 0.1 mM PMSF, and protease inhibitor cocktail]. The lysate was clarified from cellular debris by centrifugation at 30,000 × *g* for 30 min, then anti–FLAG–M2 affinity resin (Sigma–Aldrich) was added to the supernatant and carefully rotated for 2 h at 4°C. The resin was collected and washed with HMM–buffer [10 mM MOPS–NaOH (pH 7.3), 5 mM MgCl_2_, 0.1 mM EGTA, 0.1 mM DTT, 0.1 mM PMSF] completed with 500 mM NaCl and 2 mM ATP, then with low salt HMM–buffer containing 100 mM NaCl. Recombinant protein complex was eluted with 0.2 mg/mL FLAG peptide and dialyzed against HMM–buffer completed with 100 mM KCl and 1 mM DTT. The protein was used immediately or supplemented with 10% trehalose, flash–frozen in liquid nitrogen and stored at −80°C. Protein content of the final sample was proven by Western blot using Anti–Flag (F1804, Sigma–Aldrich), Anti–CaM (Abcam), Anti–Myl12b (sc–28329, Santa Cruz Biotechnology) and Anti–Myl6 (PA5–106803, Thermo Fisher Scientific) primer antibodies.

Human Tpm1.6 (NCBI Reference Sequence NP_001018004.1), Tpm1.8 (NCBI Reference Sequence NP_001288218.1) and Tpm3.1 (NCBI Reference Sequence NP_705935.1) were produced tag–free in *E. coli* strain Rosetta^™^ (Merck) (Pathan–Chhatbar et al., 2018; Reindl et al., 2022). After overproduction in *E. coli*, the lysate [containing 50 mM Hepes (pH 7.5), 200 mM NaCl, 5 mM MgCl_2_, 5 mM DTT, 0.5 mg/mL lysozyme and protease inhibitor] were heated up to 80°C for 10 min and then cooled on ice. The soluble fraction was filtered, Tpm was precipitated at the appropriate pI, separated by centrifugation and resuspended in low salt buffer [20 mM Tris–HCl (pH 7.2), 100 mM NaCl, 5 mM MgCl_2_] for anion exchange chromatography. The fractions containing Tpm was concentrated by precipitation. The protein was supplemented with 3% sucrose, flash–frozen in liquid nitrogen and stored at −80°C (Pathan–Chhatbar et al., 2018; Reindl et al., 2022). Human β–actin (Uniprot–ID: P60709) was produced in *Sf9* cells as described previously (Greve et al., 2024). α–skeletal actin (Uniprot–ID: P68139) was prepared from chicken pectoralis major muscle, as previously described for rabbit α–skeletal actin (Lehrer and Grace, 1972).

### Steady–state ATPase

Steady–state kinetics were performed at 30°C with the NADH–coupled assay in a buffer containing 20 mM MOPS–NaOH (pH 7.3), 5 mM MgCl_2_, 1 mM EGTA, 0.5 mg/mL phosphoenolpyruvate, 50 μg/mL pyruvate kinase, 20 μg/mL lactate dehydrogenase, 1 mM NADH, 2 mM ATP, 2 mM DTT. The myosin concentration was 0.15 µM. The RLC of Myo5c–HMM (3LC) was phosphorylated immediately prior to use at 25°C for 30 min 2 μM Myo5c–HMM (3LC) was incubated with recombinant human myosin light chain kinase (MLCK, Merck KGaA, Darmstadt, Germany) at a stoichiometric ratio of 20:1 in HMM–buffer containing 0.2 µM CaM, 1 mM CaCl_2_, 1 mM ATP and 1 mM DTT. The ATPase rate was normalized to the Myo5c–HMM concentration and was plotted against the F–actin concentration. The F–actin concentration was varied between 0 and 40 µM. NADH oxidation was followed using the change in the absorption at 340 nm (ε = 6220 M^−1^ cm^−1^) in a temperature–controlled plate reader (Multiskan FC, Thermo Fisher Scientific Inc. Waltham, MA, United States) using UV–transparent microtiter plates. The ATPase rate was normalized to the Myo5c–HMM concentration. The ATPase activity in the absence of F–actin was subtracted from the actin–activated data. ATPase rate pro motor domain was plotted against the F–actin concentration and nonlinear curve fitting was performed using Origin 2022b (OriginLab Corporation, Northampton, MA, United States) statistical program. The parameters k_cat_, K_app.actin_, and k_cat_/K_app.actin_ were obtained by fitting the data to the Michaelis–Menten equation. K_0.5_ defines K_app.actin_, plateau values define k_cat_, and k_cat_/K_app.actin_ is defined by the initial slope of the fit curve at concentrations of actin much lower than K_app.actin_. To investigate the influence of Tpm, F–actin was preincubated with 1.3–fold molar excess of the different Tpm isoforms for 30 min at room temperature. To study the inhibition of Myo5c by PBP, Myo5c–HMM was preincubated for 20 min at room temperature with different concentrations of PBP (Agarwal and Knölker, 2004; Fedorov et al., 2009).

### *In vitro* motility assay

*In vitro* motility assay using Atto550–phalloidin–labelled actin filaments and surface–immobilized Myo5c–HMM were performed as previously described with minor modifications (Reindl et al., 2022). Briefly, flow cells used in the *in vitro* motility assay were constructed using nitrocellulose–coated coverslips. 0.1 mg/mL Myo5c–HMM diluted in assay buffer [25 mM MOPS–NaOH (pH 7.4), 50 mM KCl, 5 mM MgCl_2_, 10 mM DTT] was flushed into the flow cell. To prevent unspecific binding of actin to the coverslip surface, 0.5 mg/mL bovine serum albumin was flushed into the flow cell and incubated for 3 min. Next, 20 nM Atto550–phalloidin (Merck) labelled F–actin was added and incubated for 3 min. Motility was started by the addition of 4 mM ATP in assay buffer completed with 0.5% methylcellulose and an anti–photobleaching mix (5 mg/mL glucose, 100 μg/mL glucose–oxidase, 100 μg/mL catalase). Image series were recorded at an Olympus IX70 inverted fluorescence microscope (Olympus, Hamburg, Germany) equipped with a 60×/1.49 NA PlanApo oil immersion objective and an Orca Flash 4.0 CMOS camera (Hamamatsu Photonics Deutschland GmbH, Herrsching, Germany). At least three video sequences were recorded in each experiment. Sliding velocity and filament length distribution of at least 300 actin filaments were analyzed with FiJi software using the user modified wrMTrck plugin (Schindelin et al., 2012; Nussbaum–Krammer et al., 2015).

### *In silico* modelling

A structural model of the motor domain of human Myo5c (Uniprot–ID: Q9NQX4) was generated with AlphaFold2 version 2.3.1, the fasta file was accessed on 1 November 2023 (Jumper et al., 2021). Different conformers and tautomers of PBP [2,3,4–tribromo–5–(3,5–dibromo–2–hydroxyphenyl)–1H–pyrrole] were generated with QM Conformer and Tautomer Predictor of Jaguar (Jaguar, Schrödinger, LLC, New York, NY, 2023). PBP was inserted, with Glide, into the model of human Myo5c (Glide, Schrödinger, LLC, New York, NY, 2023.). The glide grid was generated in accordance to the position of PBP in the structure of the motor domain of *Dictyostelium discoideum* myosin–2 (PDB–ID: 2JHR). The PDB–structure was accessed on 11 October 2023. Analysis of the structural models and visualization of the results was done with Maestro in the Schrödinger Suite 2023–4 (Maestro, Schrödinger, LLC, New York, NY, 2023). The structure was prepared with the Protein Preparation Workflow and the minimization was done with the Minimization tool of Maestro (Protein Preparation Wizard; Epik, Schrödinger, LLC, New York, NY, 2023) for 200 cycles with 80 steps per cycle in the VSGB solvate model (Li et al., 2011). ΔG was calculated with the Prime MM–GBSA v3.000 tool for the minimized structure (Prime, Schrödinger, LLC, New York, NY, 2023).

### Immunohistochemistry

We used the following species–specific antibodies directed against epitopes in the Myo5c heavy chain. The rat polyclonal antibody PA5–65120 recognizes sequences in the C–terminal part of the coiled–coil forming region of the human Myo5c heavy chain. The rabbit polyclonal antibody PA5–103939 is directed against sequences of the rat Myo5c heavy chain that are thought to correspond to IQ6 and the adjacent hinge residues. Both antibodies were supplied by Thermo–Fisher^®^; they were used at a dilution of 1:400. The guinea pig polyclonal antibody IR00261–2 was obtained from Agilent Dako^®^ and used at a dilution of 1:300. The antibody cross–reacts with high specificity with insulin from several mammalian species, including human and rat insulin. The mouse monoclonal antibody (F8/86): sc–53466, raised against von Willebrand factor protein (VWF) of human origin, was supplied by Santa Cruz Biotechnology, Inc.^®^ and used at a dilution of 1:200. DAPI was used for nuclear counterstaining. Studies on the specificity of the primary antibodies were performed by omission of the primary or secondary antibodies labelled with different fluorescent dyes resulting in the loss of an immunostaining in the different sections. Tissue specimens were fixed in 4% paraformaldehyde in 0.15 mol/L PBS and embedded in paraffin. The human tissues of non–diabetic and type 1 diabetic subjects obtained by organ resection during surgical intervention or from organ donors have been approved by the responsible Pisa and Hannover ethics committees. Tissues of non–diabetic and diabetic rats from the human Type 1 diabetes animal model, the LEW.1AR1–iddm rat, bred in the Central Animal Facility of Hannover Medical School according to the principles of laboratory care have been approved by the Lower Saxony State Office (AZ: 2014/56).

## Results

### Myo5c colocalizes with insulin in pancreatic β–cells

**FIGURE 1:**
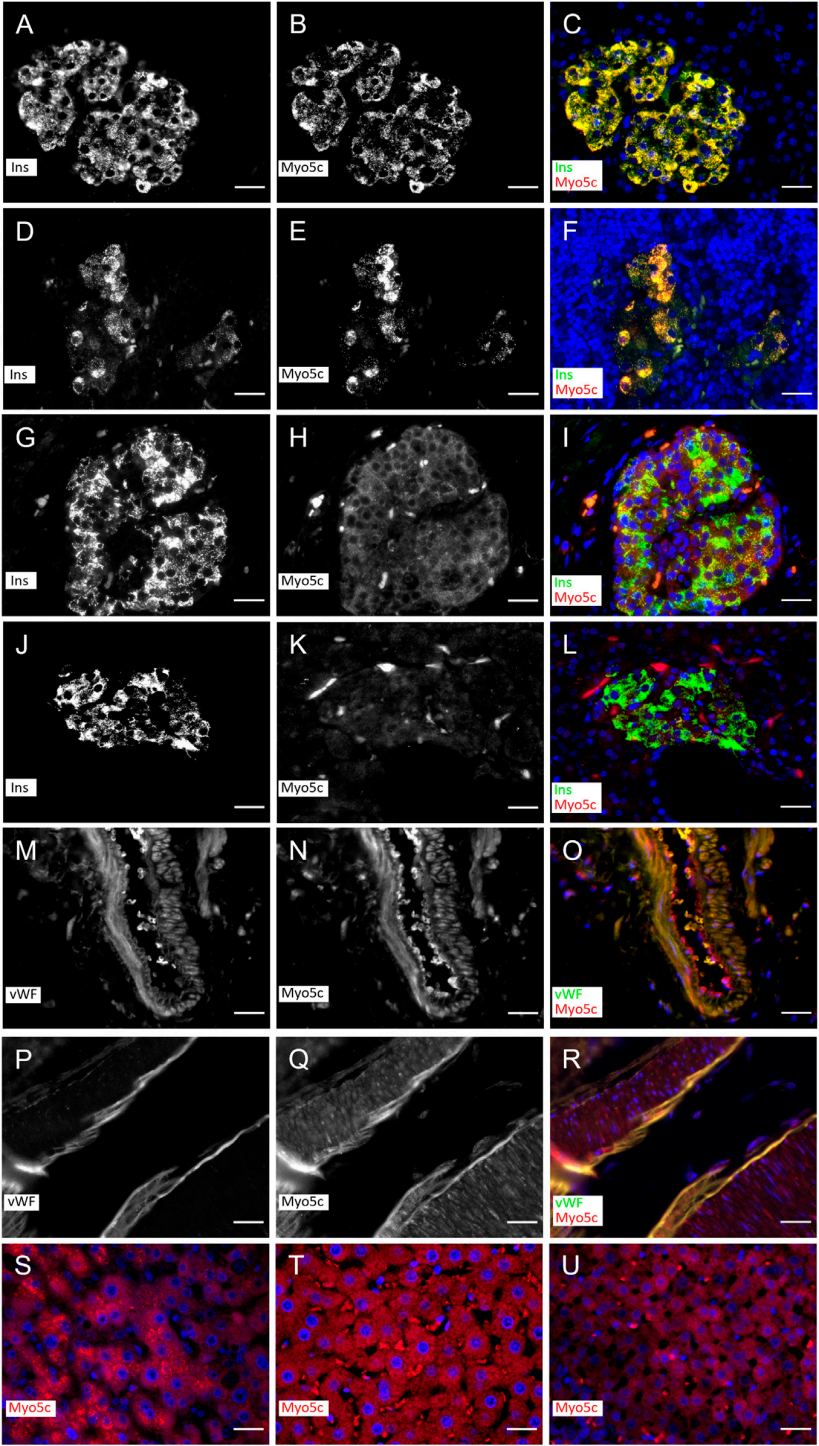
Myo5c immunostaining in β–cells in islets of rat **(A–F)** and human **(G–L)** under non–diabetic and diabetic conditions and endothelial cells of vessels of rat **(M–O)** and human **(P–R)**. Myo5c is colocalized with insulin (Ins) staining resulting in an orange–yellow overlay in the β–cells under non–diabetic **(A–C,G–I)** and diabetic **(D–F,J–L)** conditions. The endothelial layer of vessels shows a Myo5c expression in colocalization with the von Willebrand factor (vWF) **(M–O,P–R)**. A moderate protein expression of Myo5c is found also in hepatocytes of human **(S)** and rat **(T)** as well as in endocrine cells of the rat adrenal cortical region **(U)**. Nuclei are stained with DAPI. Scale Bar 25 µm.

Myo5c is abundant in the pancreas. Previous studies have shown that Myo5c appears in the exocrine part of the tissue, where it is localized to epithelial and endothelial cells (Rodriguez and Cheney, 2002; Holthenrich et al., 2022). In this study, we utilized tissue sections to investigate additional aspects of Myo5c localization. Immunostaining of human and rat pancreatic islets revealed Myo5c in insulin–producing beta cells, showing strong colocalization with insulin staining under normoglycemic and hyperglycemic conditions after autoimmune diabetes development (Figures 1A–L). In addition, strong Myo5c staining and strong colocalization with von Willebrand factor was observed in the endothelia of small to large pancreatic vessels in both species (Figures 1M–R). Moderate levels of Myo5c were found in human and rat hepatocytes and in the cortical region of rat adrenal glands (Figures 1S–U).

### Myo5c binds RLC, ELC and CaM

**FIGURE 2:**
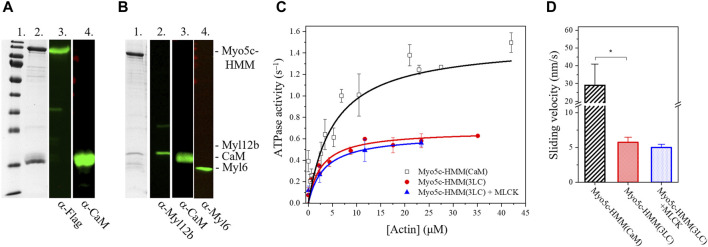
Myo5c–HMM co–produced with CaM, RLC, and ELC light chains has lower motor activity as co–produced with CaM only. **(A)** Purified Myo5c–HMM co–produced with CaM light chain. (*Lane 1*) MW marker. (*Lane 2*) Coomassie gel showing the result of purification via Flag affinity chromatography. Blot of Myo5c–HMM stained with (*Lane 3*) anti–Flag or (*Lane 4*) anti–CaM. Marker bands from highest to lowest (180, 130,100, 70, 55, 40, 35, 25, 15, 10 kDa). **(B)** Purified Myo5c–HMM co–produced with CaM, RLC, and ELC light chains. (*Lane 1*) Coomassie gel showing the result of purification via Flag affinity chromatography. Blot of Myo5c–HMM stained with (*Lane 2*) anti–Myl12b, (*Lane 3*) anti–CaM or (*Lane 4*) anti–Myl6. **(C)** Steady–state actin–activated ATPase activity of human Myo5c coproduced with CaM only (black squares) or with CaM, RLC and ELC (red circles) at various F–actin concentration ranging between 0 and 45 µM. RLC was phosphorylated by MLCK (blue triangles). **(D)** Average sliding velocity of actin over the Myo5c–HMM–coated surface, obtained from *in vitro* motility assay, was determined from at least 300 individual trajectories (mean ± SD). RLC was phosphorylated by MLCK. Asterisk marks significance *p* < 0.01.

The different human myosin V isoforms are commonly produced with CaM as a light chain in recombinant systems (Takagi et al., 2008; Heissler et al., 2017). We successfully produced and purified human Myo5c–HMM in the presence of CaM [Myo5c–HMM (CaM)], ∼0.5 mg protein could be produced from 2 L *Sf9* cell pellet (Figure 2A). However, recent data suggests that unconventional myosins may use other possible light chains beside CaM, such as the RLCs, ELCs, or other calmodulin–like proteins (Bird et al., 2014; Holló et al., 2023). To investigate this, *Sf9* cells were coinfected with three baculovirus vectors containing the Myo5c–HMM heavy chain, CaM and the RLC/ELC (Myl12b/Myl6). Myo5c was successfully co–produced and co–purified with different light chains. Immunoblot analysis using antibodies against the different light chains demonstrated that CaM, RLC and also ELC was bound to the heavy chain (Figure 2B). The purification of Myo5c–HMM containing all three light chains [Myo5c–HMM (3LC)] from 2 L *Sf9* cell pellet resulted in ∼0.5 mg protein, and less degradation was observed during and after the purification. Based on the Coomassie–stained gel, the binding ratio between the light chains and heavy chain supposed to be around 3:1 for RLC, 2:1 for CaM and 1:1 for ELC. Meanwhile, Myo5c–HMM binds six CaM, when coproduced with CaM only (Supplementary Figure S1).

### Phosphorylation by MLCK has no effect on the motor function of Myo5c

RLC phosphorylation is a key regulatory mechanism of non–muscle myosin II isoforms (Garrido–Casadoet al., 2021). The motor domain is primarily activated by phosphorylation of Ser19 of the RLC by the calcium/CaM dependent MLCK. Phosphorylation enhances the ATPase activity ∼25–fold (Sellers and Harvey, 1984; Vicente–Manzanares et al., 2009). In this study, we assessed whether RLC phosphorylation affects the ATPase activity of Myo5c. Before conducting the NADH–coupled ATPase assay, Myo5c–HMM was pre–incubated with MLCK and CaM for 20 min at 20°C in a Ca^2+^–containing buffer. We observed no significant increase in ATPase activity, either in the basal or actin–activated state, suggesting that phosphorylation of Ser19 of the RLC has no effect on Myo5c–HMM activity. However, at 0.67 ± 0.01 s^−1^, the k_cat_ measured for Myo5c–HMM (3LC) with or without phosphorylation is only about half the value measured for Myo5c–HMM (CaM) (Figure 2C).

An actin-activated ATPase activity alongside successful filament gliding confirms both the enzymatic capability and the motor function of myosin. ATPase activity indicates that myosin can bind and hydrolyze ATP, which is essential for its function as a motor protein. In addition, if performed in the presence of a range of actin concentrations, the assay provides information about the efficiency of coupling between the actin and nucleotide binding sites in the myosin motor domain. However, it doesn’t necessarily prove that the motor can function correctly in terms of movement or force generation. The filament gliding assay confirms that the myosin can convert chemical energy (ATP) into mechanical work (movement) by utilizing actin as a track. Therefore, combining both assays gives a more complete assessment of myosin motor function, providing complementary insights into their enzymatic activity and ability to generate force and movement.

To further test the motor function and the possible regulation of Myo5c, *in vitro* motility assay was performed, in which Myo5c–HMM was pre–phosphorylated by MLCK. The sliding velocity measured for Myo5c–HMM (3LC) was significantly lower than that of Myo5c–HMM (CaM). Phosphorylation by MLCK did not significantly change the sliding velocity of Myo5c–HMM, which is consistent with the results obtained from the ATPase assay (Figure 2D). These results indicate that phosphorylation by MLCK does not affect the motor function of Myo5c–HMM.

### Tpm isoform has an impact on the motor function of Myo5c

Cytoskeletal tropomyosin isoforms play a crucial role in regulating myosin motor function (Pathan–Chhatbar et al., 2018; Reindl et al., 2022). In this study, we investigated the effect of Tpm3.1, 1.6 and 1.8 isoforms on the motor function of Myo5c–HMM (CaM) and Myo5c–HMM (3LC).

**FIGURE 3:**
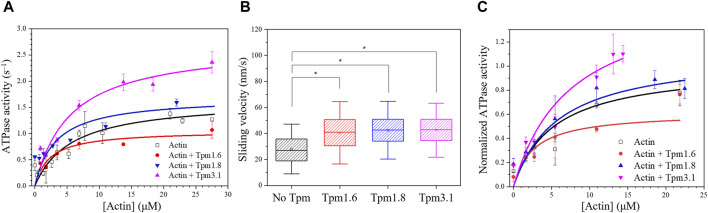
Tropomyosin isoforms affect the motor properties of Myo5c–HMM. **(A)** Steady-state actin-activated ATPase activity of human Myo5c–HMM (CaM) at various F-actin concentration ranging between 0 and 27 μM, in the presence of different Tpm isoforms. Tpm was added in a 1.3–fold molar excess, measurements were performed at 30°C (*n* = 2, mean ± SE). **(B)** Box chart showing the results of *in vitro* motility assay. Average sliding velocity of actin or actin–Tpm complex over the Myo5c-HMM (CaM)–coated surface was determined from at least 300 individual trajectories. 10 μM Tpm was added to all buffer solutions, assays were performed at 25°C. Box width shows percentile 25–75, whiskers represent ±SD. Asterisks mark significance *p* < 0.01. **(C)** Tpm isoforms have an impact on the motor function of Myo5c–HMM (3LC). Data were normalized to the k_cat_ of the actin–activated ATPase activity of Myo5c–HMM (3LC) in the absence of Tpm, measurements were performed at 30°C (*n* = 3, mean ± SE).

The steady–state actin–activated ATPase activity of Myo5c–HMM (CaM) was measured in the presence of either bare actin or Tpm–actin cofilaments (Figure 3A). The maximal rate of ATP hydrolysis (k_cat_) was determined to correspond to 1.65 ± 0.14 s^−1^ in the absence of Tpm. K_app.actin_, the substrate concentration required for half–maximal activity, which gives an approximation of the actin affinity of Myo5c in the presence of ATP, was found to be 5.7 ± 1.45 µM.

Measurements performed with actin–Tpm cofilaments revealed significant changes in k_cat_, K_app.actin_ or k_cat_/K_app.actin_. In the presence of Tpm3.1, we observed a 1.6–fold increase in k_cat_ from 1.65 ± 0.14 s^−1^ to 2.71 ± 0.28 s^−1^. Cofilaments with Tpm1.6 gave a 35% reduction in k_cat_, while cofilaments with Tpm1.8 gave a 43% reduction in K_app_ from 5.70 ± 1.45 µM to 3.24 ± 1.58 µM. The apparent second order rate constant (k_cat_/K_app.actin_), which represents the coupling efficiency between the actin and nucleotide binding sites, was increased approximately 1.5–fold for all three Tpm isoforms. The kinetic data are summarized in Table 1.

**TABLE 1:** Impact of Tpm isoforms on the kinetic behavior of Myo5c–HMM (CaM).

Substrate	k_cat_ (s^−1^)	K_app actin_ (µM)	k_cat_/K_app actin_ (µM^−1^s^−1^)	Sliding velocity (nm/s)
Actin	1.65 ± 0.14	5.70 ± 1.45	0.29 ± 0.09	28.68 ± 12.45
Actin + Tpm1.6	1.05 ± 0.11	2.31 ± 0.88	0.45 ± 0.13	40.53 ± 15.93
Actin + Tpm1.8	1.69 ± 0.21	3.24 ± 1.58	0.52 ± 0.13	41.42 ± 14.76
Actin + Tpm3.1	2.71 ± 0.28	5.54 ± 1.72	0.48 ± 0.16	41.71 ± 13.82

*In vitro* motility assays were performed to further investigate the effects of the Tpm isoforms on the motility of Myo5c. The *in vitro* sliding velocity of bare actin filaments was 29 ± 12 nm/s. The sliding velocity of fluorescently labeled and phalloidin–stabilized actin filaments on surfaces coated with Myo5c–HMM (CaM) was approximately 45% faster following preincubation of actin filaments with an excess of Tpm isoforms Tpm3.1, 1.6 or 1.8 (Figure 3B; Table 1).

The presence of Tpm1.6, 1.8 and 3.1 in cofilaments with actin induced distinct changes in Myo5c–HMM (3LC) motor function. Tpm3.1 increased the ATP turnover of Myo5c–HMM (3LC) by about 60%, while Tpm1.6 decreased it by about 40% (Figure 3C).

### Myo5c motor function is inhibited by PBP

**FIGURE 4:**
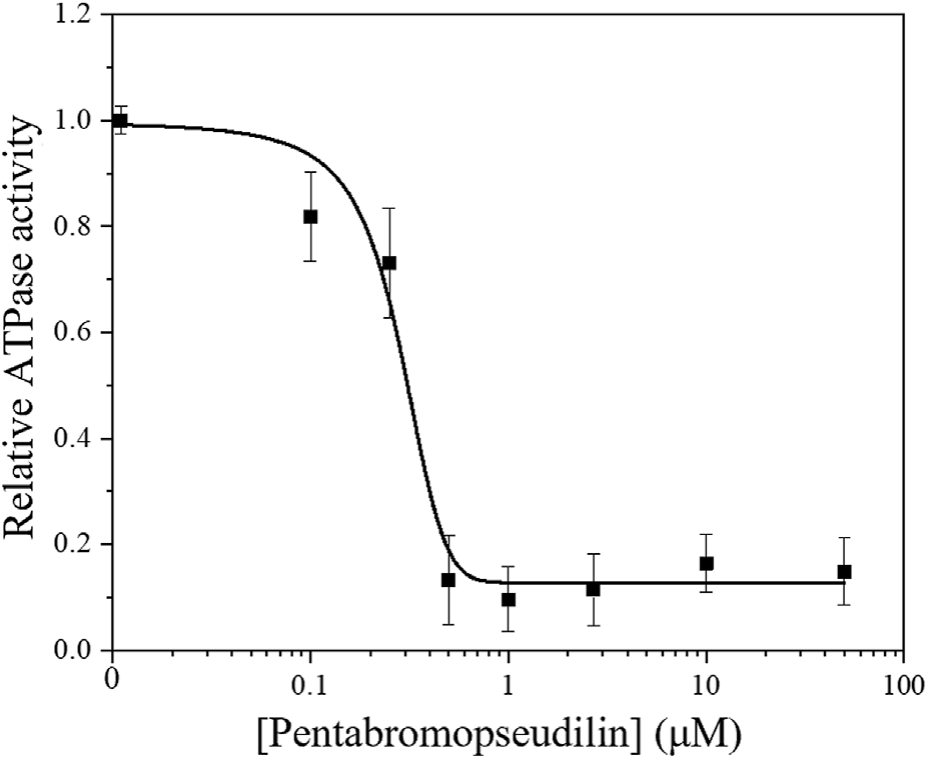
Steady–state actin–activated ATPase activity of human Myo5c–HMM at 13 μM F–actin concentration, in the presence of 0–50 µM PBP. The semilogarithmic plot shows the concentration dependence of Myo5c–HMM inhibition. The data were fitted with a dose-response model, using the equation y = A_1_ + (A_2_−A_1_)/{1 + 10^[(LOGx0−x)*P]^}, where the coefficient *P* is an empirical parameter reflecting the allosteric nature of the mechanism of PBP-mediated inhibition. The concentrations of PBP required for half–maximal inhibition (IC_50_) of Myo5c–HMM was determined from the fit (*n* = 3, mean ± SE).

Small allosteric effectors can interfere with the mechanochemical cycle and either enhance or inhibit myosins (Manstein and Preller, 2020). The halogenated pseudilin, PBP was identified as potent inhibitors of class V myosins: the ATPase activity of *D. discoideum* Myo5b and chicken Myo5a is greatly reduced in the presence of PBP (Fedorov et al., 2009). Based on the homology within the myosin V class it is assumed that PBP is likely to be an effective inhibitor of human Myo5c. Therefore, we assessed the effect of increasing concentrations of PBP on the ATPase activity of our Myo5c–HMM construct. In the presence of 13 µM actin, the ATPase activity of Myo5c–HMM decreased in a sigmoidal manner with increasing PBP concentration (Figure 4). The half–maximal inhibitory concentration was determined to be 280 ± 60 nM, obtained from a dose–response fit for the entire data set.

### Docking model of PBP to Myo5c predicts strong binding

We prepared an AlphaFold2 model of the motor domain of human Myo5c. The model is in the pre–power stroke conformation, similar to the model of the motor domain of Myo2 from *D. discoideum*, which was crystallized in the presence of PBP and Mg^2+^–ADP–meta–vanadate. In the *Dd* Myo2 structure, the PBP binding pocket is formed by residues from helix 13 (Lys265–Val268), helix 21 (Val411–Leu441), the strut loop (Asn588–Gln593) and loop 2 (Asp614–Thr629) (Fedorov et al., 2009; PDB–ID: 2JHR). The residues involved in PBP binding are only moderately conserved between the members of the different myosin classes, as reflected by the observed differences in the IC_50_ values for myosin isoforms from class I, II and V, and the results of molecular modeling studies probing the interactions of PBP with *Dd* Myo1E, *Sc* Myo2, *Gg* Myo5a, and *Dd* Myo5b. *Gg* Myo5a showed in these experiments the strongest inhibition by PBP of ATP binding, ATP turnover and motor activity. Consistent with these results, the number and strength of predicted interactions with PBP were greatest for *Gg* Myo5a (Fedorov et al., 2009).

**FIGURE 5:**
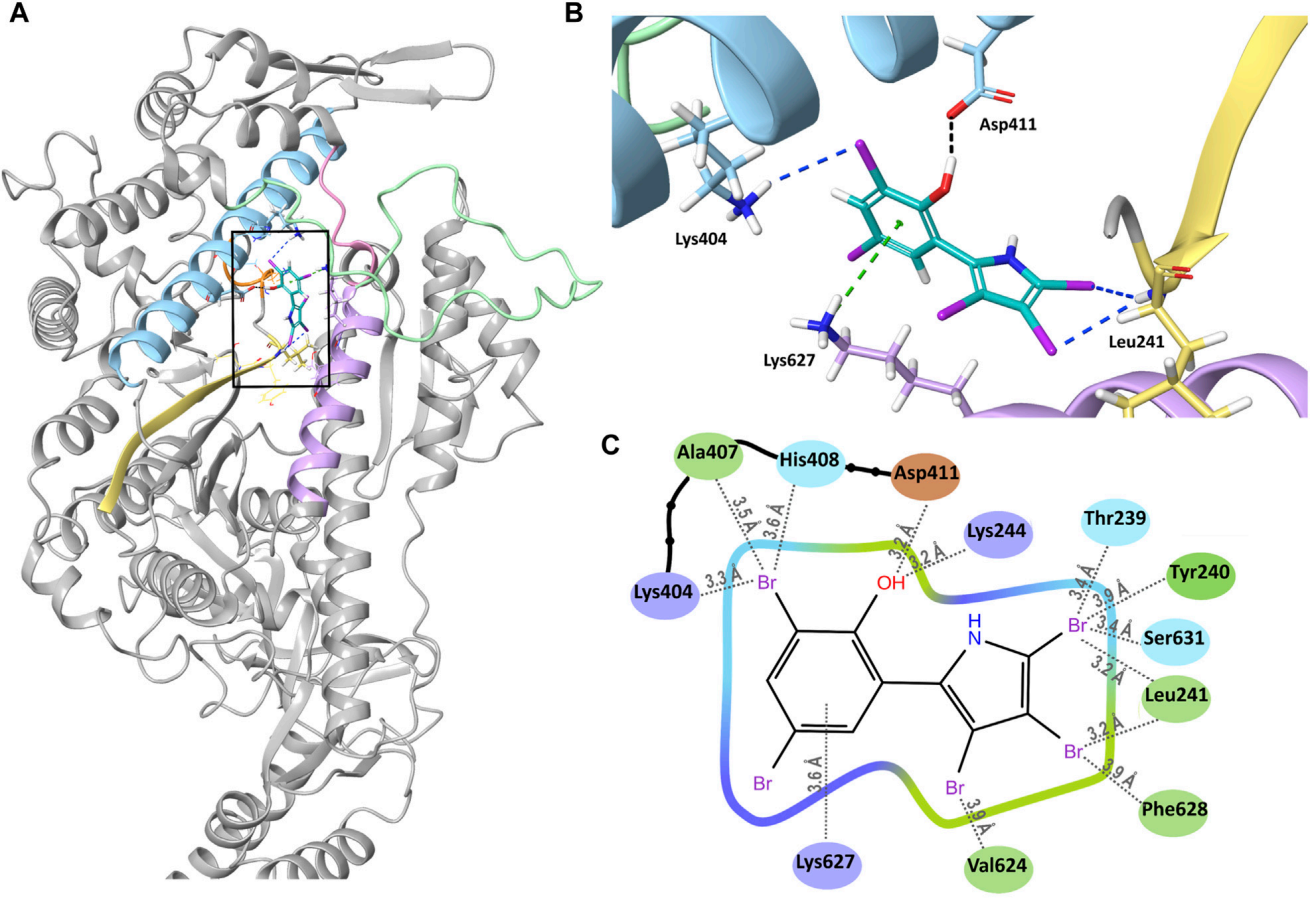
Binding of PBP to Myo5c. **(A)** Overview of PBP (cyan) bound into its binding groove (black square) in Myo5c. Helix 13 (Lys244–Val247) is shown in orange, Helix 21 (Arg391–Leu421) in blue, the strut loop (Asn566–Tyr571) in pink, loop 2 (Asn588–Thr623) in green, helix 29 (Val624–Asn640) in violet and the β–strand connecting β7 and helix 13 in yellow. **(B)** Close–up of the PBP binding site, shown from the direction of the back of the head rotated 90° to the right from the overview position. Hydrogen bond (black), halogen bonds (blue) and cation–pi interaction (green) between PBP and Myo5c are shown, structures of Myo5c in the same colors as in **(A)**. **(C)** Ligand Interaction Diagram of PBP and Myo5c residues within 4 Å. Residues are colored by their properties: negative residues in orange, positive residues in violet, hydrophobic residues in green and polar residues in blue. Distances to the nearest atom are shown with grey dotted lines.

We docked PBP to our AlphaFold2 model of Myo5c, using the Glide docking application of Schrödinger. We created different conformers and tautomers of PBP and selected the most chemically stable ones for docking. After minimization, we selected the PBP conformer with the strongest bonds. PBP forms several interactions in the binding groove (Figure 5A). The phenyl ring forms a pi–cation interaction with Lys627 of helix 29. The oxygen of the phenyl ring forms hydrogen bond with Asp411 of helix 21. Leu241 (β–strand connecting ß7 and helix 13) forms halogen bonds with Br3 and Br4 of the pyrrole ring. Lys244 of helix 13 forms a halogen bond with Br3 of the phenyl ring (Figures 5B, C). The calculated binding energy (ΔG) of this interaction is with −18.44 kcal/mol comparable of the binding energy reported for the interaction of PBP with *Gg* Myo5a (Fedorov et al., 2009).

## Discussion

The physiological role and regulation of Myo5c, the most recently discovered member of the class V myosins, are still not fully understood. However, its abundance in the majority of the tissues (except in the neural and in some lymphatic tissues) suggests its significance. It has the highest expression level among the myosin V isoforms in epithelial and glandular tissues (Rodriguez and Cheney, 2002).

This study demonstrates that recombinant human Myo5c–HMM can be co–produced and co–purified not only with the conventionally accepted CaM, as light chain of class V myosins, but also with the RLC Myl12b and ELC Myl6. This follows a recent trend in literature where different unconventional myosins were successfully purified with light chains other than CaM, such as RLC, ELC or CALML4, which often represents a more effective purification process, resulting in a more stable protein. For example, Myo15 binds CaM, RLC (Myl12b) and ELC (Myl6) as light chains (Bird et al., 2014). Mouse Myo5a or *Drosophila* Myo7a can be purified exclusively with CaM, in contrast, Myo5a purified from chicken brain was shown to be associated with CaM and ELC and human Myo7a with CaM, RLC (Myl12b) and CALML4 (Yang et al., 2006; Heissler and Sellers, 2014; Holló et al., 2023). These findings suggest that various light chains may be physiologically relevant, but also that they may be species–specific.

When Myo5c–HMM is co–produced with CaM alone in the baculovirus/*Sf9* system, we assume that each of the six IQ motifs of Myo5c has CaM bound to it. In the presence of all three light chains, we observed light chain:heavy chain ratios of 3:1 for RLC, 2:1 for CaM and 1:1 for ELC. The enzymatic activity of Myo5c appears to be modulated by the type of light chains bound. The maximal actin–activated ATPase activity of Myo5c–HMM (3LC) was only about half of that co–produced exclusively with CaM Myo5c–HMM (CaM) and the actin sliding velocity was also significantly lower. The activity of Myo5c has not previously been shown to be modulated by the type of light chains bound.

An important regulatory mechanism of the RLC– and ELC–containing class II myosins is the phosphorylation of the Ser19 and Thr18 amino acids of the RLC, which enhances the ATPase activity (Sellers, 1985). We tested the possible effect of phosphorylation on Myo5c when co–produced with RLC, but no difference was observed in the ATPase activity or actin sliding velocity after phosphorylating the RLC with calcium/CaM dependent MLCK. This suggests that the RLC–containing construct may not be fully active or that another regulatory mechanism is required, such as phosphorylation of the RLC on a different amino acid or by another kinase.

The regulation of unconventional myosins by different light chains is an interesting topic. For example, Myo5a exhibit kinetic differences, depending on whether it is coproduced with CaM or different ELC isoforms (La et al., 2000). Although CaM, RLC, or ELC are abundant in most tissues, there is limited knowledge about the *in vivo* exchange of light chains. The observed variations in light chain composition during myosin isolation (Wang et al., 2000), or the successful experiments using different light chains with the same myosin, suggest that unconventional myosins may be functional with different sets of light chains. However, further examination is necessary to confirm this possibility.

The motor activity of Myo5c–HMM, regardless of the type of the light chain was influenced by different Tpm isoforms. Tpm isoforms are important regulators of the actomyosin cytoskeleton, affecting the function of various myosins. Tpm3.1, for example, enhances the motor function of non–muscle Myo2a and is a more favorable track for Myo5a compared to bare actin filament (Sckolnick et al., 2016; Reindl et al., 2022). Actin filaments in complex with Tpm3.1 appear to be a more effective substrate for Myo5c–HMM both in steady–state ATPase and *in vitro* motility assays, by increasing the coupling efficiency between the actin and the nucleotide binding site on the myosin head. All the examined Tpm isoforms, including Tpm3.1, Tpm1.6, and Tpm1.8, increase the sliding velocity of actin filaments on a Myo5c–HMM covered surface and show increased coupling efficiency. However, the long Tpm1.6 isoform decreases the maximal ATPase activity of Myo5c–HMM, while Tpm1.8 does not cause any significant change. As discussed in Reindl et al. (2022), Tpm sequences contributing to the overlap region are not the sole determinant of differences in actin affinity. Instead, global sequence differences between Tpm isoforms are of greater importance than variable exon 6 usage, which leads to the diversity in the mechanical properties of various actin–Tpm cofilaments. Furthermore, cryo-EM studies have shown that Myo2c interacts directly with Tpm3.1 through the loop 4 of the motor domain in the acto–myosin–Tpm complex (von der Ecken et al., 2015). As this region is not particularly conserved, further molecular docking simulations are required to determine the interacting structures between Myo5c and the various Tpm isoforms.

Several physiological and pharmaceutical factors can influence the motor function of a myosin. Small allosteric effectors, which alleviate certain steps of the kinetic cycle of a motor protein, are promising candidates in drug development, but also useful tools in myosin research (Manstein and Preller, 2020). PBP, a marine–derived polybrominated pseudilin was originally developed as an anti–cancer drug candidate, blocking the enzymatic activity of human lipoxygenase. Later it was found to interfere with the motor function of class I, II and V myosins. The strongest inhibition was observed for chicken acto–Myo5a, with a half–maximal dose of 1.2 ± 0.2 µM and as low as 0.4 ± 0.2 µM in the absence of actin, which was more than a magnitude lower than that for the other myosin isoforms (Fedorov et al., 2009). To examine the inhibitory effect of PBP on human Myo5c, we conducted a steady–state actin–activated ATPase assay. The inhibitory effect on acto–Myo5c–HMM was more pronounced, with an IC_50_ as low as 280 ± 60 nM. This result was supported by *in silico* analysis. Molecular docking simulations predict that PBP binds to Myo5c in a groove near the actin and nucleotide binding sites, similar to previously described models for *Dd* Myo2 and *Gg* Myo5 (Fedorov et al., 2009; Behrens et al., 2017). The binding energy of *Hs* Myo5c to PBP is ∼30–times higher than that of *Dd* Myo2, which is consistent with the difference in the inhibitory effect observed in the steady–state actin–activated ATPase assay.

Our *in silico* data show that the binding energy between *Hs* Myo5c and PBP is almost the same as that between Gg Myo5a and PBP. In analogy to *Gg* Myo5a, an even more pronounced effect of PBP is expected in the absence of actin. This would result in an even lower IC_50_ for human Myo5c. Our prediction suggests a potential binding between PBP and Myo5c, however, it is important to note that this is only a prediction. To obtain more detailed information about the binding mechanism, additional molecular dynamics simulations, and crystallization experiments are required. Our results confirm previous findings that PBP is not strictly class–specific but acts preferentially on class V myosins.

A prominent localization of Myo5c was found in the pancreas. Earlier observations showed Myo5c to localize with low abundance to the apical region of the acinar cells of the exocrine pancreas in mice (Rodriguez and Cheney, 2002). Our findings, based on the use of human and rat tissues, show the presence of Myo5c in the endocrine pancreas. Specifically, we observed a strong colocalization of Myo5c with insulin in β–cells. Following the manifestation of autoimmune diabetes in the pancreas, when insulin production is severely reduced by the loss of β–cells in the pancreatic islets, Myo5c immunofluorescence was correspondingly reduced. In pancreatic β–cells, insulin–containing dense core vesicles (secretory granules) are transported from the endoplasmic reticulum to the Golgi compartment and then intracellularly to various cell domains for storage and to the cell periphery for secretion by exocytosis. This translocation is fine-tuned by the cooperative work of different motor proteins. The budding of the proinsulin containing secretory granules from the trans–Golgi network is mediated by Myo1b (Tokuo, Komaba, and Coluccio, 2021). The transport of the secretory granules occurs primarily along microtubules using kinesin motors, while closer to the cortex the vesicles are moved along actin tracks using myosin V motors (Varadi et al., 2002). Previous studies have shown that Myo5a participates in the transport of insulin secretory granules by attaching to the dense core vesicles through granulophilin–a, a Rab27a effector protein (Wang et al., 1999). Other members of the small GTPase Rab family are also involved in recruiting Myo5a to the vesicle surface. Rab3a associates specifically with synaptic vesicles and insulin secretory granules (Regazzi et al., 1996). The absence of Rab3a in mice was shown to lead to severe hyperglycemia and insulin secretory deficiencies (Varadi, Tsuboi, and Rutter, 2005). Rab3a is likely to bind to the convex region of the GTD of Myo5a and Myo5c, but not Myo5b, according to recent molecular docking data. Additionally, the myosin binding site on Rab3a differs from that of rabphilin–3a, an effector protein of Rab3a (Dolce et al., 2020). Given that Myo5c is the most prominent myosin V isoform in the pancreas and is able to bind Rab3a, it appears likely that Myo5c may act synergistically with Myo5a in insulin secretion. Furthermore, it has been demonstrated in mouse pancreatic tissue that Tpm3.1 may have a role in regulating insulin secretion. Treatment of pancreatic β–cells with TR100 or ATM1001 anti–Tpm compounds resulted in a reduction of glucose-stimulated insulin secretion, meanwhile the impact was significantly less in Tpm3.1 KO mice compared to WT (Kee et al., 2018).

The consequences of depleting Myo5c in pancreatic islets have not yet been studied. It is worth noting, that Myo5c is a non–processive motor, unlike the processive Myo5a or Myo5b, however, it can move processively in an ensemble or on actin bundles (Krementsova et al., 2017). This process may be influenced by Tpm isoforms, providing favorable or unfavorable tracks for vesicular transport. Future research will focus on how Myo5c–actin–tropomyosin complexes can be used as therapeutic targets to modulate insulin secretion.

## Data Availability

The datasets presented in this study can be found in online repositories. The names of the repository/repositories and accession number(s) can be found in the article/Supplementary Material.

## Abbreviations

Myo5c, myosin 5c; PBP, pentabromopseudilin; CCD, coiled–coil doman; GTD, globular tail domain; CaM, calmodulin; RLC, regulatory light chain; ELC, essential light chain; Tpm, tropomyosin; MLCK, myosin light chain kinase.
