# Supplementary material for: Motor properties of Myosin 5c are modulated by tropomyosin isoforms and inhibited by pentabromopseudilin
Source: Front Physiol. 2024 Mar 28;15:1394040. doi: 10.3389/fphys.2024.1394040 (PMC11008601; doi:10.3389/fphys.2024.1394040)
Supplement: Supplementary file 1 [file DataSheet1.PDF]

Supplementary Material

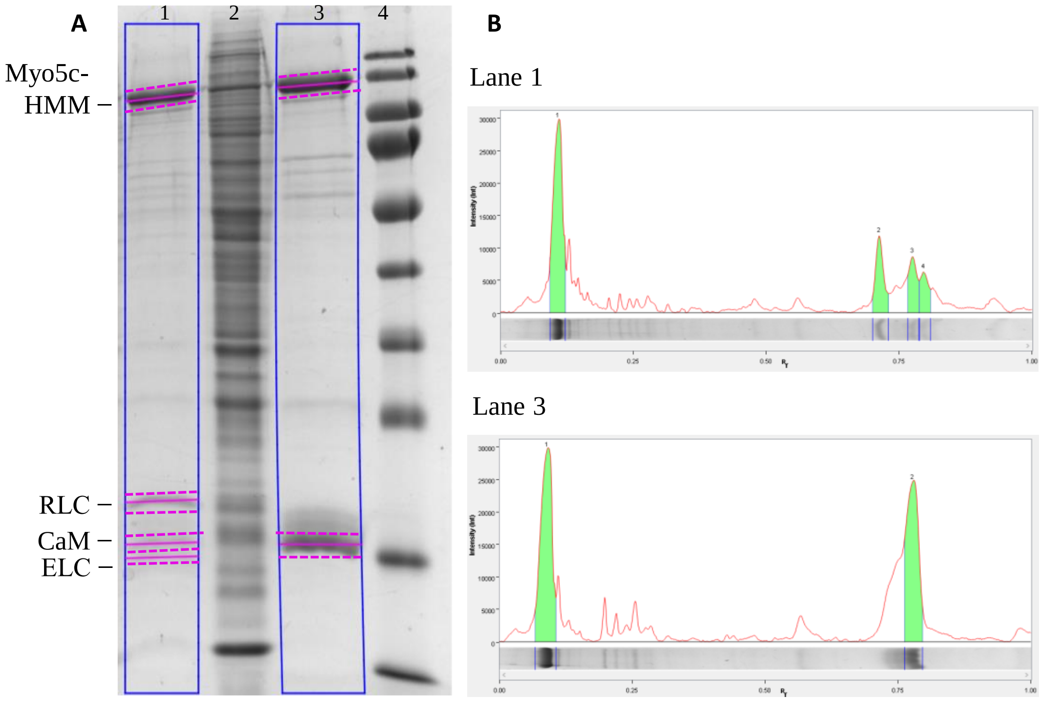

C

		Volume (Int)	MW (kDa)	Rel.Quant/ MW	**LC/HC**	
Lane 1	HC	49635906	128.994	384792		
	RLC	17673976	19.779	893570	**2.32**	**RLC/HC**
	CaM	13378155	16.837	794568	**2.06**	**CaM/HC**
	ELC	7993212	16.93	472133	**1.23**	**ELC/HC**
Lane 3	HC	66042486	128.994	511981		
	CaM	56048334	16.837	3328879	**6.50**	**CaM/HC**

Supplementary Figure 1. Calculation of light chain / heavy chain ratio (LC/HC) in the different Myo5c samples, using Image Lab 6.0.1 (Bio-Rad Laboratories, Inc.). (**A**) Commassie gel, *Lane 1.* Myo5c-HMM(3LC) with the adjusted boundaries, *Lane 2.* Sf9 cell suspension infected with Myo5c-HMM P2 baculovirus, *Lane 3.* Myo5c-HMM(CaM) with the adjusted boundaries, *Lane 4.* MW Marker (Marker bands from highest to lowest: 180, 130 100, 70, 55, 40, 35, 25, 15, 10 kDa). (**B**) Intensity profile of *Lane 1* and *Lane 3*. (**C**) Table shows the adjusted volume of the proteins in the bands (the background adjusted intensities within the boundaries), which were normalized to the molecular mass and stochiometric ratio was calculated.
